# The judgement of biases included in the category “other bias” in Cochrane systematic reviews of interventions: a systematic survey

**DOI:** 10.1186/s12874-019-0718-8

**Published:** 2019-04-11

**Authors:** Andrija Babic, Andela Pijuk, Lucie Brázdilová, Yuliyana Georgieva, Marco António Raposo Pereira, Tina Poklepovic Pericic, Livia Puljak

**Affiliations:** 1 Institute of Emergency Medicine in Split-Dalmatia County, Split, Croatia; 2 Medical student, University of Split School of Medicine, Split, Croatia; 3 Faculty of Medicine and Dentistry, Palacký University Olomouc, Olomouc, Czech Republic; 4 Medical University of Plovdiv, Plovdiv, Bulgaria; 5 Faculty of Health Sciences - University of Beira Interior, Covilhã, Portugal; 6 Catholic University of Croatia, Zagreb, Croatia

**Keywords:** Systematic review, Cochrane, Risk of bias, Other bias inconsistency

## Abstract

**Background:**

Clinical decisions are made based on Cochrane reviews, but the implementation of results of evidence syntheses such as Cochrane reviews is problematic if the evidence is not prepared consistently. All systematic reviews should assess the risk of bias (RoB) in included studies, and in Cochrane reviews, this is done by using Cochrane RoB tool. However, the tool is not necessarily applied according to the instructions. In this study, we aimed to determine the types of bias and their corresponding judgements noted in the ‘other bias’ domain of Cochrane RoB tool.

**Methods:**

We analyzed Cochrane reviews that included randomized controlled trials (RCTs) and extracted data regarding ‘other bias’ from the RoB table and accompanying support for the judgment. We categorized different types of other bias.

**Results:**

We analyzed 768 Cochrane reviews that included 11,369 RCTs. There were 602 (78%) Cochrane reviews that had ‘other bias’ domain in the RoB tool, and they included a total of 7811 RCTs. In the RoB table of 337 Cochrane reviews for at least one of the included trials it was indicated that no other bias was found and supporting explanations were inconsistently judged as low, unclear or high RoB. In the 524 Cochrane reviews that described various sources of other bias, there were 5762 individual types of explanations which we categorized into 31 groups. The judgments of the same supporting explanations were highly inconsistent. We found numerous other inconsistencies in reporting of sources of other bias in Cochrane reviews.

**Conclusion:**

Cochrane authors mention a wide range of sources of other bias in the RoB tool and they inconsistently judge the same supporting explanations. Inconsistency in appraising risk of other bias hinders reliability and comparability of Cochrane systematic reviews. Discrepant and erroneous judgments of bias in evidence synthesis may hinder implementation of evidence in routine clinical practice and reduce confidence in otherwise trustworthy sources of information. These results can help authors of Cochrane and non-Cochrane reviews to gain insight into various sources of other bias that can be found in trials, and also to help them avoid mistakes that were recognized in published Cochrane reviews.

**Electronic supplementary material:**

The online version of this article (10.1186/s12874-019-0718-8) contains supplementary material, which is available to authorized users.

## Background

Assessment of the risk of bias (RoB) in included studies is an integral part of preparing Cochrane systematic reviews. Bias is any systematic error that can negatively affect the estimated effects of interventions and lead authors to wrong conclusions about efficacy and safety of analyzed interventions [1].

Cochrane reviews use Cochrane’s RoB tool, whose aim is to enable better appraisal of evidence and ultimately lead to better healthcare [2]. Cochrane’s standard RoB tool has seven domains. First domain addresses random sequence generation as a potential source of selection bias, assessing potentially biased allocation to interventions due to inadequate generation of a randomized sequence. Second domain analyzes allocation concealment, which can also lead to selection bias. The third domain is devoted to blinding of participants and personnel; it is associated with performance bias due to the knowledge of the allocated interventions by participants and personnel during the study. Fourth domain addresses blinding of outcome assessment; if done inadequately, it can lead to detection bias due to the knowledge of the allocated interventions by outcome assessors. Fifth domain analyzes the presence of incomplete outcome data, which can yield attrition bias due to amount, nature or handling of incomplete outcome data. The sixth domain is devoted to selective reporting, which can cause reporting bias due to selective outcome reporting. And finally, there is the seventh domain of Cochrane RoB assessment called “other bias”, which is used to note bias occurring due to any additional problems that were not covered by the first six domains [3].

The Cochrane Handbook provides some examples of other potential threats to validity, such as design-specific risk of bias in non-randomized trials, baseline imbalance between groups of participants, blocked randomization in trials that are not blinded, differential diagnostic activity, study changes due to interim results, deviations from the study protocol, giving intervention before randomization, inappropriate administration of an intervention or having co-intervention(s), contamination due to drug pooling among participants, insufficient delivery of intervention, inappropriate inclusion criteria, using instruments that are not sensitive for specific outcomes, selective reporting of subgroups and fraud [3].

This list of potential other sources of bias mentioned in the Cochrane Handbook is limited, and it would, therefore, be useful to explore potential additional sources of ‘other bias’. By consulting a more comprehensive list of potential other biases, the systematic review might recognize certain problems in included studies that might not otherwise consider a potential source of bias.

The aim of this study was to define which issues authors of Cochrane reviews describe as “other bias”, to determine the prevalence of various categories of other bias and to quantify qualitative data which support the assessment of other bias.

## Methods

We conducted a retrospective analysis of published Cochrane reviews.

### Inclusion and exclusion criteria

We retrieved Cochrane reviews that included RCTs about interventions published from July 2015 to June 2016 (*N* = 955) by using Advanced search in The Cochrane Library. Diagnostic Cochrane reviews, empty reviews, overviews of systematic reviews and reviews withdrawn in this period were excluded. Cochrane reviews that included both RCTs and non-randomized trials were included, but only RoB of RCTs were analyzed.

### Screening

One author assessed all titles/abstracts to establish the eligibility of Cochrane reviews for inclusion (LP). Another author verified all the assessments of the first author (AB). There were no disagreements.

### Data extraction and categorization

Data extraction table was developed and piloted using five Cochrane reviews. Initially, one author manually extracted the data by copy-pasting from included Cochrane reviews and another author verified 10% of extractions. Of the 77 verified Cochrane reviews, we found 3 Cochrane reviews which were partially extracted (3.9%), which we consider to be a negligible percentage of the discrepancy. We extracted judgments (high, low or unclear risk) and supporting explanations for judgments (qualitative data which support the assessment to determine the reasons for the judgment) from the ‘other bias’ section of RoB table in Cochrane reviews. We also extracted judgments and support for judgments from additional non-standard domains (domains which are not covered by seven standard RoB domains in RoB table mentioned in the Background section) if Cochrane authors used them. For Cochrane reviews that did not use the ‘other bias’ domain in the RoB table or any other additional non-standard domains, we analyzed the text of results to see whether Cochrane authors mentioned any potential sources of other bias in the text of the review only. Each supporting explanations for judgments of risk of bias in the analyzed trials were categorized by two authors (AB and LP), via consensus. In 2018 we enlisted a help of information specialist who used software for data extraction, and compared manually extracted data with software-extracted data; we found 12 further discrepancies in extracted judgments.

### Outcomes

We analyzed number, type, judgments and inconsistencies in judgments for certain comments about other risk of bias. These inconsistencies were judged as follows: we analyzed whether Cochrane authors used different RoB judgments for the same supporting comment. We quantified Cochrane reviews in which authors did not use ‘other bias’ domain for any of the included RCTs to determine whether they used some non-standard additional RoB domain instead of ‘other bias’. We conducted a quantitative and qualitative analysis of these non-standard domains.

### Statistics

We performed descriptive statistics using Microsoft Excel (Microsoft Inc., Redmond, WA, USA). We presented data as frequencies and percentages. In the primary analysis, we analyzed Cochrane reviews that had the ‘other bias’ domain in the RoB table. In the secondary analysis, we analyzed Cochrane reviews that did not have the ‘other bias’ domain or had different non-standard variations of RoB assessment that were not mentioned in the Cochrane Handbook.

## Results

### Primary analysis

**Fig. 1:**
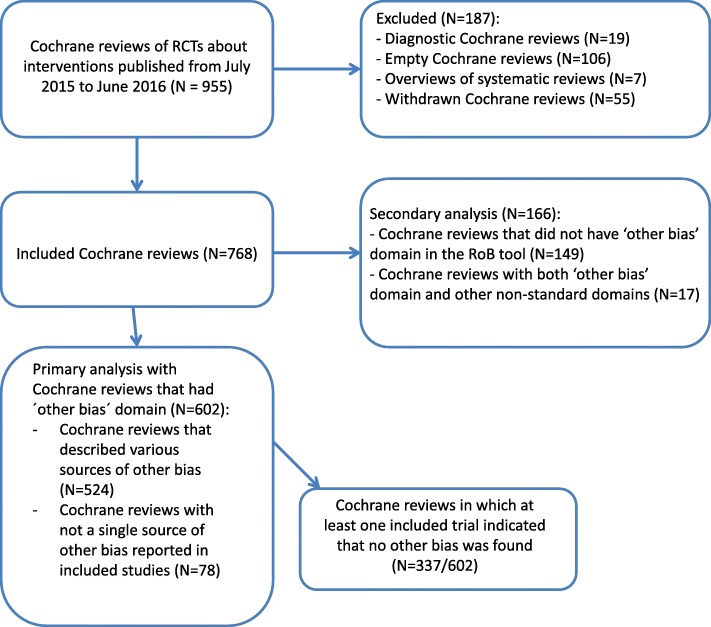
Flow diagram presenting the inclusion of Cochrane systematic reviews in the study. We retrieved 955 Cochrane systematic reviews from the Cochrane Database of Systematic Reviews that were published from July 2015 to July 2016. We excluded 187 Cochrane reviews because they were either empty (without a single study included), diagnostic accuracy reviews, overviews of systematic reviews or they were withdrawn. We included 768 Cochrane reviews in our analysis; of those, 602 were included in our primary analysis because they had other bias domain in the Cochrane risk of bias tool, while 166 Cochrane reviews were included in our secondary analysis because they either did not have other bias domain in the Cochrane risk of bias tool, or they had this domain, but also other non-standard domains in the tool

We analyzed 768 Cochrane reviews that included 11,369 RCTs. Among those 768 Cochrane reviews, we included in the primary analysis 602 Cochrane reviews that had ‘other bias’ domain in the RoB tables. Those 602 Cochrane reviews included a total of 7811 RCTs. We analyzed 166 Cochrane reviews in the secondary analysis because they either did not have ‘other bias’ domain in RoB Tables (*N* = 149), or those Cochrane reviews had both ‘other bias’ domain and additional non-standard domains in the RoB Tables (*N* = 17). The flow diagram showing inclusion of Cochrane reviews is shown in Fig. 1.

Out of 602 Cochrane reviews in the primary analysis, there were 524 (87%) Cochrane reviews that described various sources of bias in the ‘other bias’ domain, while in 78 (13%) Cochrane reviews not a single source of other bias was reported. Furthermore, among 602 Cochrane reviews from the primary analysis, there were 337 (56%) Cochrane reviews in which at least one included trial indicated that no other bias was found. Terminology for comments about non-existent other bias varied, even within individual Cochrane reviews. In 268 (80%) Cochrane reviews only one version of the comment that no other bias was found was used, while in 69 (20%) reviews Cochrane authors used different expressions in comments to indicate that no other sources of bias were found. Some examples of this varied terminology are shown in Additional file 1: Table S1.

In 40 (12%) out of 337 Cochrane reviews that indicated that no other bias was found, we observed discrepancies in judgment for this domain. Namely, Cochrane authors in these 40 Cochrane reviews sometimes indicated that lack of other bias was associated with low RoB, and sometimes they marked it as unclear or high RoB. In 59 (18%) of these 337 Cochrane reviews at least one support for judgment that indicated that no other bias was identified Cochrane authors judged as not being the low risk of bias (either high or unclear); in 278 Cochrane reviews this was judged as low RoB.

In 19 Cochrane reviews, all comments that referred to no other bias being identified were judged as unclear. In one review comment, ‘no other bias’ was judged as both low and high. References to Cochrane reviews for these specific examples are in Additional file 2: Table S2. In one review the same comment was judged in different RCTs as either low or high. In one review the same comment was judged in different RCTs as either low or unclear or high.

Of the 7811 trials that were included in the 602 Cochrane reviews from the main analysis, in 3703 (47%) trials domain for other bias indicated in the support for judgment that other bias was not identified. Of those 3703 trials, there were 288 (7.8%) that were judged as unclear RoB, 4 (0.1%) that were judged as high RoB, while the others (*N* = 3411, 92.1%) were judged as low RoB.

#### Sources of other bias

In the 524 analyzed Cochrane reviews that described various sources of other bias, there were 5762 different supporting explanations for judgments of other bias that we categorized into 31 categories. In 535 trials it was indicated only that it was not possible to assess other bias. For 24 (4%) of those 535 trials it was not indicated why this was not possible, while the most common reasons for not being able to assess other bias were that there was ‘insufficient information’ (*N* = 392, 73%), the trial was published as a conference abstract only (*N* = 78, 15%) and that the trial was published in a foreign language so there were issues with translation (*N* = 11, 2%). Cochrane authors were not consistent in judging this type of supporting explanation; for 11 (2%) trials it was judged as high RoB, for 520 (94%) as unclear RoB and for 4 (0.7%) as low RoB.

There were 236 trials for which Cochrane authors simply wrote that issues related to other bias were not described or unclear. This type of supporting explanation was also inconsistently judged by the Cochrane authors; 7 (3%) judged it as low RoB and 229 (97%) as unclear RoB.

The remaining 4991 explanations for judgments of other bias were divided into 29 categories that are shown in Table 1. The most frequently used categories of explanations for other bias were related to baseline characteristics of participants, funding of a trial, reporting, sample size and conflict of interest (Table 2). Cochrane authors used the domain for other bias to indicate positive, negative and unclear aspects of a trial. For example, three most common types of explanations in the category related to baseline characteristic of participants indicated that either baseline characteristics were similar, or that there was the imbalance in baseline characteristics, or that there was insufficient information about it (Additional file 3: Table S3). Among 4991 explanations, we were unable to categorize 85 of them because they were uninformative, including explanations such as ‘Adequate’ or ‘N/A’ or ‘Other risk of bias was possible’. Finally, there were 112 explanations that were used only once or twice in RoB tables we analyzed so we categorized that group as ‘Other explanations’. A table with all the types of explanations is presented in Additional file 3: Table S3.

**Table 1:** Different categories of other bias (based on 4991 explanations) in Cochrane systematic reviews

Category	N (%)
Baseline characteristics of participants	1067 (21.4)
Funding	774 (15.6)
Sample size	405 (8.1)
Reporting	381 (7.6)
Conflict of interest	288 (5.8)
Inclusion and exclusion criteria	197 (3.9)
Confounding	196 (3.9)
Analyses	191 (3.8)
Outcome domains and outcome measures	135 (2.7)
Co-interventions	134 (2.7)
Deviations from the protocol	123 (2.5)
Randomisation	111 (2.2)
Terminated early	108 (2.2)
Issues related to cross-over trials	98 (2)
Intention-to-treat analysis (ITT)	95 (1.9)
Study design	76 (1.6)
Compliance	72 (1.4)
Attrition	71 (1.4)
Contamination	65 (1.3)
Follow-up and study duration	46 (0.9)
Blinding	25 (0.5)
Clustering	17 (0.3)
Selection bias	17 (0.3)
Protocol registration	16 (0.3)
Study quality	9 (0.2)
Publication bias	7 (0.1)
Adequacy of comparators	5 (0.1)
Inexplicable	85 (1.7)
Other	177 (3.6)

**Table 2:** Judgments for the 20 most common explanations of other bias

Explanation	Total	High, N (%); n^a^	Unclear, N (%); n^a^	Low, N (%); n^a^
Not possible to assess other bias	504	7 (1.4);*7*	494 (98);*117*	3 (0.6);*3*
Baseline characteristics similar between the groups	314	0 (0);*0*	24 (8);*13*	290 (92);*61*
Not described/unclear	233	0 (0);*0*	226 (97);*54*	7 (3);*4*
Baseline imbalance between groups of participants	167	91 (54);*56*	62 (37);*41*	14 (9);*12*
Funding: industry	162	83 (51);*28*	77 (48);*25*	2 (1);*2*
Potential confounding factors	120	63 (53);*38*	47 (39);*34*	10 (8);*9*
Not enough information on baseline characteristics of participants	88	8 (9);*6*	78 (89);*39*	2 (2);*2*
Funding: non-profit	86	0 (0);*0*	4 (5);*4*	82 (95);*33*
Funding: not reported	72	0 (0);*0*	68 (94);*15*	4 (6);*4*
Important parameters not reported	61	19 (31);*14*	41 (68);*28*	1 (1);*1*
Sample size: calculation of sample size not provided	42	24 (57);*6*	17 (41);*7*	1 (2);*1*
Potential randomisation problem	40	9 (23);*9*	28 (70);*13*	3 (7);*3*
Potential problem with inclusion criteria	40	16 (40);*15*	22 (55);*12*	2 (5);*2*
Deviations from the study protocol	37	16 (43) *13*	18 (49) *15*	3 (8) *3*
No relevant subgroup analysis	36	10 (28);*1*	26 (72);*1*	0 (0);*0*
Funding: intervention supplied by industry	32	14 (44);*7*	12 (38);*10*	6 (18);*3*
Adequate	28	0 (0);*0*	0 (0);*0*	28 (100);*1*
No information on the validity of the outcome measure	27	3 (11);*3*	23 (85);*5*	1 (4);*1*
Sample size: performed calculation	24	1 (4);*1*	3 (12);*3*	20 (84);*9*
Sample size: small	23	8 (35);*5*	15 (65);*5*	0 (0);*0*

^a^*n* = Number of Cochrane reviews that included at least one RCT with this characteristic

#### Partial studies included in the primary analysis

We found 34 Cochrane reviews with specific partial data regarding other bias, i.e. whose ‘other bias’ domains in RoB tables were not complete. We divided them into four distinct groups: the first group with 28 reviews that had judgments for ‘other bias’, but not all had accompanying comments, second group with 4 reviews where only one included RCT did not have the ‘other bias’ domain, third group with one review with included RCT without ‘other bias’ domain and included RCT with only judgment without comment, and fourth group with one review where RoB table was completely missing for 6 included RCTs. References to Cochrane reviews and RCTs for these specific examples are in Additional file 2: Table S2. Some Cochrane reviews had additional non-standard RoB domains, separately or in addition to the ‘other bias’ domain. Categories of additional non-standard RoB domains in Cochrane reviews are shown in Table 3.

**Table 3:** Categories of additional non-standard RoB domains in Cochrane systematic reviews

Additional category	N of Cochrane reviews
Group similarity at baseline (selection bias)	11
Baseline data	5
Baseline outcome measures (similar)	3
Groups balanced at baseline/ balance in baseline characteristics	2
Baseline characteristics of participants	1
Baseline comparability of treatment and control groups	1
Baseline measures	1
Similarity of baseline characteristics^a^	1
Treatment/control groups comparative at entry	1
Major imbalance in important baseline confounders	1
Comparability of groups on different prognostic characteristics^a^	1
Size	8
Size of the study	5
Small sample size bias	4
Sample size^a^	2
Sufficient sample size^a^	1
Power calculation^a^	1
Timing of outcome assessment (similar)^a^	10
Adequate follow-up	2
Study duration	2
Early stopping	1
Groups received comparable treatment	2
Care program identical/ identical care	2
Treatment fidelity^a^	1
Free of systematic differences in care?^a^	1
Consistency in intervention delivery	1
Equality of treatment	1
Protocol deviation balanced	1
Groups received same intervention	1
Compliance/adherence assessed (acceptable)	7
Compliance with recommendation reliable?	1
Compliance acceptable^a^	1
Source of funding/ sponsorship	4
For profit funding^a^	1
Funding^a^	1
Vested interest bias	1
Conflict of interest	1
Co-intervention avoided or similar^a^	5
Co-interventions	2
Groups received same co- interventions	1
Intention to treat	5
Incorrect analysis	1
Results based on data dredging?	1
Analyses adjust for different lengths of follow-up workers?	1
Appropriate statistical tests use?	1
Adequate adjustment for confounding in the analyses?	1
Contamination/ protection against contamination	3
Validity of outcome measures	1
Reliability of outcome measures	1
Outcome measures used valid and reliable?	1
Free from performance bias	1
Performance bias as «differential expertise» bias	1
Performance bias as comparability in the experience of care providers	1
Adequate patient description	1
Recruitment of participants from the same population?	1
Recruitment of participants over the same study period?	1
Washout/ carry-over effect in cross-over study designs	2
Overall assessment of bias risk	1
Summary of risk of bias for Consumption outcome	1
Researcher allegiance^a^	1
Therapist allegiance^a^	1
CHBG (Cochrane hepato-biliary group) combined assessment (mortality)^a^	1
CHBG combined assessment (hepatic encephalopathy)^a^	1
Comparability with individually randomized trials	1
Detection bias (biochemical validation of smoking outcomes)	1
Ethical approval	1
Explicit inclusion/exclusion criteria	1
Free of dietary differences other than fat?^a^	1
Loss of clusters	1
Methods for selecting cases to adjudicate	1
Outcome description	1
Publication format	1
Recruitment bias	1

^a^domains found in 9 Cochrane reviews that had both ‘other bias’ domain and additional non-standard domain(s) for other bias in RoB tables

#### Cochrane authors’ judgments of different explanations for ‘other bias’

There were 3033 trials for which only one category of explanation was written by Cochrane authors. When the explanation had only one category of comment we could be certain that the judgment referred only to that specific comment so we analyzed those in detail to see how the Cochrane authors judge different explanatory comments. There were 259 types of different explanations among those 3033 trials. We analyzed in more detail those judgments for 20 most common explanations of other bias and found very high inconsistency in how Cochrane authors judge the same explanations (Table 2).

### Secondary analysis

#### Reviews without ‘other bias’ domain in the RoB table

Among 149 Cochrane reviews that did not have ‘other bias’ domain in the RoB table, there were 102 reviews that did not have any other replacement domain for ‘other bias’. These 102 reviews used the varied number of standard RoB domains. In those 102 reviews, the number of standard RoB domains that were used varied, with one standard RoB domain in 4 reviews, three RoB domains in 7 reviews, four RoB domains in 15 reviews, five domains in 51 reviews and 6 domains in 25 reviews.

For this group of Cochrane reviews, that did not have the ‘other bias’ domain in the RoB table, we analyzed texts of results to see whether they mentioned any other sources of bias, beyond the standard six domains, in the section ‘Risk of bias in included studies’. We found that 68/102 (67%) did not mention any sources of other bias in the results of the review. However, the remaining 34 (33%) did have comments about the other bias. Three of those 34 stated that they had not found any other risk of bias, while 31 reviews out of those 34 reported in the text of results that the included studies had had from 1 to 6 different categories of other bias.

#### Reviews with both ‘other bias’ domain and additional non-standard domain(s) for other bias in RoB tables

Nine Cochrane reviews had both ‘other bias’ domain and additional non-standard domain(s) for other bias in RoB tables (References in Additional file 2: Table S2). Those reviews used from 1 to 4 additional non-standard domains; 18 in total. Those additional non-standard RoB domains are listed in Table 3 and marked with the asterisk.

#### Reviews without ‘other bias’ domain but with the additional non-standard domain(s)

There were 57 Cochrane reviews that did not have the ‘other bias’ domain, but they did have additional non-standard RoB domains apart from the standard domains in the Cochrane RoB table. Most of the reviews had only one additional non-standard domain (*N* = 24), while others had 2–8 additional domains per each RCT. Table 3 shows non-standard domains that were used in those reviews without ‘other bias’ domain.

#### Reviews that consistently did not use support for judgment or they used non-standard judgments

We found 9 Cochrane reviews that consistently did not use supporting explanations for judgment or they used non-standard judgments. In 5 reviews authors used judgments low, high or unclear RoB, but without comments as support for judgment. In one review all trials were marked with the unclear risk of other bias without any comment as support for judgment. In four reviews all trials were marked with low risk of other bias without any comment as support for judgment. We also found 4 reviews that did not have judgments low-high-unclear, but different kinds of judgments. One review had judgments yes/no without supporting comments; two reviews had judgments yes, no or unclear, with supporting comments and there was one review with judgments A-adequate and B-unclear (References in Additional file 2: Table S2).

## Discussion

In this study, we analyzed 768 Cochrane systematic reviews, with 11,369 included trials. We found that Cochrane authors used numerous different categories of sources of other bias and that they were not judging them consistently. We categorized different types of supporting explanations into 31 categories, and we found numerous other inconsistencies in reporting of sources of other bias in Cochrane reviews. Findings of this study are disconcerting because consistency in secondary research is very important to ensure comparability of studies.

Insufficient and unclear reporting of the ‘other bias’ domain was very common in the Cochrane reviews we analyzed. Among the most common support for judgment were comments that we categorized as ‘not described/unclear’, which is puzzling because ‘other bias’ domain is not specific like the other six domains of the RoB tool, and it is, therefore, difficult to fathom what it means that other bias was not described or that it was unclear. If the authors did not find sources of other bias, or if they thought that they could not assess other bias because of the brevity of report or language issues, they should have stated that. Likewise, for some trials, the only supporting explanation was that other bias was ‘Adequate’. Without any further explanations, readers cannot know what exactly the Cochrane authors found to be adequate in terms of other potential sources of bias. Many systematic reviews had a high number of included studies, and therefore some comments were repeated multiple times in the same systematic review.

The most commonly used specific category of other bias referred to baseline characteristics of participants. In RCTs, randomization should ensure allocation of participants into groups that differ only in intervention they received. Randomization should ensure that the characteristics of participants that may influence the outcome will be distributed equally across trial arms so that any difference in outcomes can be assumed to be a consequence of intervention [4]. Baseline imbalances between the groups may indicate that there was something wrong with the randomization process, or that they might be due to chance [5]. Severe baseline imbalances can occur because of deliberate actions of trialists if they aim to intentionally subvert the randomization process [6] or due to unintentional errors.

Chance imbalances should not be considered a source of bias, but it may be difficult to distinguish whether baseline imbalances are caused by chance or intentional actions. If there are multiple studies included in a meta-analysis, it could be expected that chance imbalances will act in opposite directions. But the problem may occur if there is a pattern of imbalances across several trials that may favor one intervention over another, suggesting imbalance due to bias and not due to chance [7]. Cochrane is now developing a second generation of the RoB tool, titled RoB 2.0, and one of the signaling questions in the RoB domain about randomization process asks “Were there baseline imbalances that suggest a problem with the randomization process” [7]. The fact that so many Cochrane authors used comments about baseline imbalance as a domain of other bias, and not in the RoB domain about random sequence generation (selection bias) indicate that many Cochrane authors consider that this aspect should be emphasized separately from the selection bias domain.

The second most commonly used category of supporting explanations was related to funding of a trial, and comments about conflicts of interest were the fifth most common category. This is in direct contrast with the recommendations from the Cochrane Handbook, where it is acknowledged that information about vested interests should be collected and presented when relevant, but not in the RoB table; such information should be reported in the table called ‘Characteristics of included studies’ [8]. RoB table should be used to describe specific methodological aspects that may have been influenced by the vested interest and directly lead to RoB [8]. Therefore, it is obvious that the authors of the Cochrane Handbook assume that the influence of sponsors can be mediated via other domains of RoB tool such as selective reporting of favorable outcomes.

However, Lundh et al. have published a Cochrane review in 2017 about industry sponsorship and research outcomes, in which they included 75 primary studies, which shows that commercial funding leads to more favorable efficacy results and conclusions compared to non-profit funding [9]. They concluded that industry sponsorship introduces bias that cannot be explained by standard domains of Cochrane’s RoB assessment [9]. The debate about whether funding presents the source of bias or not is ongoing in the Cochrane, with some considering that commercial funding is a clear risk of bias, while others argue against such standpoint [10, 11]. This debate apparently reflects the current situation in which many Cochrane authors continue to use funding and conflict of interest as a source of other bias despite the official warning against such use of information about sponsorship from the Cochrane Handbook, as we have demonstrated in this study.

The third most frequent category of supporting explanations for other bias was related to poor reporting, where Cochrane authors indicated that relevant information was missing or were inadequately reported. Poor reporting hinders transparency, as it allows authors to avoid attention to weak aspects of their studies. For this reason, reporting guidelines should be used [12].

Comments about sample size were the fourth most common category either in a sense that the trial did or did not report sample size calculation, or that sample size was “small” without any further explanation of what the Cochrane authors considered to be a small sample. There were 21 trials for which Cochrane authors wrote that there were fewer than 50 participants in each arm. It is unclear where this cut-off is coming from, as there is no such guidance in the Cochrane Handbook in the chapter about the risk of bias. On the contrary, chapter 8.15.2. of the Cochrane Handbook specifically warns that “sample size or use of a sample size (or power) calculation” are examples of quality indicators that “should not be assessed within this domain” [8].

The Cochrane Handbook also warns that authors should avoid double-counting, by not including potential sources of bias in the ‘other bias’ domain if they can be more appropriately covered by other domains in the tool [8]. As can be seen by our study, Cochrane authors sometimes do double-counting because there were categories of comments supporting judgments that could have been addressed in the first six domains.

As we have shown, most Cochrane authors decided to use the other bias domain to describe potential additional biases that were not covered in the first six domains of the RoB tool. In the proposed RoB tool 2.0 there is no ‘other bias’ domain [7]. The proposed RoB tool is much more complex, compared to the current version of the RoB tool, and many items that were specifically emphasized by Cochrane authors in the other bias domain, as shown in our study, are addressed in the RoB 2.0 tool. However, there are still potential biases from other sources that the RoB 2.0 may neglect by omitting the RoB domain for other bias. Relevant other bias that were identified in our study include, for example, problems with inclusion and exclusion criteria, data analyses, outcome domains and outcome measures that were used, usage of co-interventions that are not accounted for, deviations from the protocol, study design, issues related to specific types of trials such as cross-over trials and biases specific to other to certain topics. Therefore, we believe that there is a rationale for including ‘other bias’ domain in revised RoB tool too.

We have already conducted a similar analysis of Cochrane RoB domain related to other RoB domains, and we found that judgments and supports for judgments in those domains were very inconsistent in Cochrane reviews [13–15]. This analysis related to sources of other bias in Cochrane reviews contributes to the perception that Cochrane RoB tool is inconsistently used among Cochrane authors. The authors do not necessarily follow guidance from the Cochrane Handbook. In the support for judgment, they mention issues that the Cochrane Handbook explicitly warns against. Various comments that serve as supports for judgments were inconsistently judged across Cochrane reviews and trials included in those reviews. Cochrane authors also use inconsistent terminology to describe the same concepts. Increasing complexity of the RoB tool, as proposed in the RoB tool 2.0 will likely only increase this problem of insufficient consistency in RoB appraisal and worsen this problem of insufficient comparability of judgments of RoB across Cochrane reviews.

Furthermore, our study indicated that Cochrane authors extensively use the available option to customize the RoB table. We found that there were as many as 102 (13%) out of 768 analyzed Cochrane reviews that did not use the other bias domain in the RoB table at all. Cochrane reviews are produced using the software Review Manager (RevMan). As soon as an author inserts a new study in the RevMan among included studies, an empty RoB table for the study automatically appears, with seven pre-determined domains. Therefore, Cochrane authors need to intentionally remove or add some domains if they want to customize the RoB table. Among 102 Cochrane reviews that did not have other bias domain, 33% of those reviews had comments about other potential sources of bias in the body of the manuscript. It is unclear why some Cochrane authors use only text for comments about other bias instead of using RoB table for this purpose. Additionally, we observed that in many Cochrane reviews without other bias domain there were other customizations of the RoB table, which had from one to six other, standard RoB domains included. Exactly half of those reviews without other bias domain in the RoB table had less than six standard domains in the RoB table.

Results of this study can contribute to better reporting of future systematic reviews and help authors of systematic reviews to avoid mistakes. Firstly, results of this manuscript will provide more comprehensive information for Cochrane authors regarding ‘other bias’ domain – we present many sources of other bias that Cochrane authors recognize, and that are not mentioned in the Cochrane Handbook. Secondly, we showed mistakes that Cochrane authors are doing when they mention in ‘other bias’ domain issues that actually belong to other six domains of Cochrane RoB tool. Thirdly, we are also pointing out mistakes that Cochrane authors are doing despite explicit instructions from the Handbook, i.e. authors use sample size and funding to comment about potential bias, even though the Handbook explicitly warns against this. Although our study was focused only on Cochrane reviews, our results are relevant also for non-Cochrane reviews that use Cochrane’s risk of bias tool. Therefore, our manuscript can help authors of Cochrane and non-Cochrane reviews to create better and more consistent reviews, to recognize additional potential sources of bias in trials they analyze, and to avoid mistakes that we have observed.

Limitation of our study is that we included in our analysis a limited number of analyzed Cochrane reviews, which were published in 2015 and 2016. We chose this convenience sample of Cochrane reviews because we were interested in the state of the ‘other bias’ domain in recent times; we did not aim to analyze the change of this domain over the very long time period. However, considering the number of Cochrane reviews analyzed, and the number of inconsistencies we observed, we have no reason to suspect that the results would be significantly different if a bigger cohort of published Cochrane reviews would have been used. It takes a long time to manually extract, check, analyze and categorize more than ten thousands of RoB domains, and therefore using the same methodology on a larger sample might not be feasible. It is possible that some unintentional errors in categorizations may have been made, and therefore, for transparency, we decided to present all categories and sub-categories of the supporting explanations we encountered in the Additional files 1, 2 and 3. Additionally, all systematic reviews are not the same and our findings cannot be generalized to all systematic reviews – we analyzed only Cochrane systematic reviews of RCTs because Cochrane RoB tool was developed for these types of studies. However, we believe that our findings can be very useful also for authors of non-Cochrane reviews who will use Cochrane RoB tool in their methodology.

Finally, it is worth emphasizing that it is possible that some trials from our cohort were included in more than one review, and that Cochrane authors could give them different judgments for ‘other bias’. It has been shown before that authors of different reviews can make different RoB judgments of the same trials [16]. However, such analysis was not the aim of our study.

## Conclusion

Cochrane authors mention a wide range of sources of other bias in the RoB tool and they inconsistently judge the same supporting explanations. Inconsistency in appraising risk of other bias hinders reliability and comparability of Cochrane systematic reviews. Discrepant and erroneous judgments of bias in evidence synthesis may hinder implementation of evidence in routine clinical practice and reduce confidence of practitioners in otherwise trustworthy sources of information. These results can help authors of Cochrane and non-Cochrane reviews to gain insight into various sources of other bias that can be found in trials, and also to help them avoid mistakes that were recognized in published Cochrane reviews. Potential remedies include more attention to author training, better resources for Cochrane authors, better peer-review and editorial consistency in the production of Cochrane systematic reviews.

## Additional files

Additional file 1: **Table S1.** Some examples of different versions of support for judgment indicating that no other bias was found. In 268 (80%) Cochrane reviews only one version of the comment that no other bias was found was used, while in 69 (20%) reviews Cochrane authors used different expressions in comments to indicate that no other sources of bias were found. Some examples of this varied terminology are shown in Table S1. (DOCX 13 kb)

Additional file 2: **Table S2.** Cochrane systematic reviews and randomized controlled trials specifically mentioned in the results as those that had different judgment for having no bias, partial information about other bias, or were included in secondary analyses. In 19 Cochrane reviews, all comments that referred to no other bias being identified were judged as unclear. In one review comment, ‘no other bias’ was judged as both low and high. References to Cochrane reviews for these specific examples are shown in this Additional file. (DOCX 87 kb)

Additional file 3: **Table S3.** Categories of explanations of other bias in analyzed Cochrane risk of bias tables. In the 524 analyzed Cochrane reviews that described various sources of other bias, there were 5762 different supporting explanations for judgments of other bias that we categorized into 31 categories. The main text describes the most common categories of explanations, while all the types of explanations is presented in Table S3. (XLSX 37 kb)

## Abbreviations

RCT: Randomized controlled trial
RCTs: Randomized controlled trials
RoB: Risk of bias
