# Supplementary material for: The judgement of biases included in the category “other bias” in Cochrane systematic reviews of interventions: a systematic survey
Source: BMC Med Res Methodol. 2019 Apr 11;19:77. doi: 10.1186/s12874-019-0718-8 (PMC6458756; doi:10.1186/s12874-019-0718-8)
Supplement: Supplementary file 1 — Table S1. Some examples of different versions of support for judgment indicating that no other bias was found. In 268 (80%) Cochrane reviews only one version of the comment that no other bias was found was used, while in 69 (20%) reviews Cochrane authors used different expressions in comments to indicate that no other sources of bias were found. Some examples of this varied terminology are shown in Table S1. (DOCX 13 kb) [file 12874_2019_718_MOESM1_ESM.docx]

**Supplementary table 1. Some examples of different versions of support for judgment indicating that no other bias was found**

| There are no apparent other sources of bias |
| --- |
| No other sources of bias were identified |
| The study appeared to be free of other sources of bias |
| None obvious |
| None apparent |
| None |
| No other potential source of bias identified |
| We did not identify any other sources of bias |
| We were unable to identify any other sources of bias |
| We did not identify any other potential sources of bias identified |
| No other bias identified |
| Study appears free of other biases |
| We do not suspect other bias |
