# Supplementary material for: The judgement of biases included in the category “other bias” in Cochrane systematic reviews of interventions: a systematic survey
Source: BMC Med Res Methodol. 2019 Apr 11;19:77. doi: 10.1186/s12874-019-0718-8 (PMC6458756; doi:10.1186/s12874-019-0718-8)
Supplement: Supplementary file 2 — Table S2. Cochrane systematic reviews and randomized controlled trials specifically mentioned in the results as those that had different judgment for having no bias, partial information about other bias, or were included in secondary analyses. In 19 Cochrane reviews, all comments that referred to no other bias being identified were judged as unclear. In one review comment, ‘no other bias’ was judged as both low and high. References to Cochrane reviews for these specific examples are shown in this Additional file. (DOCX 87 kb) [file 12874_2019_718_MOESM2_ESM.docx]

**Supplementary table 2. Cochrane systematic reviews and randomized controlled trials specifically mentioned in the results as those that had different judgment for having no bias, partial information about other bias, or were included in secondary analyses**

*Reviews that had different judgments for having no bias*

In one Cochrane systematic reviews having no other bias was judged as both low and high [1]. In one Cochrane review the same comment was judged in different randomized controlled trials (RCTs) as either low or high [2]. In one Cochrane review the same comment was judged in different RCTs as either low, or unclear or high [3].

*Partial studies included in the primary analysis*

We found 34 Cochrane reviews with specific partial data regarding other bias. We divided them into four distinct groups. In the **first group** there were 28 Cochrane reviews which included RCTs that had judgments for 'other bias', but no accompanying comments [4-31].

In the **second group** there were 4 Cochrane reviews where only one included RCT does not have 'other bias' domain [32-35]. In three Cochrane reviews one RCT did not have 'other bias' domain. For two of these studies [32, 34] this resulted in blank space for this domain in the Figures 2 and 3, which depict summary of risk of bias (RoB) across domains and studies. However, for the one Cochrane review [35], even though 'other bias' domain was missing from the RoB table for one RCT [36], in the Figures 1 and 2, the 'other bias' domain was present as “unclear”. One [33] Cochrane review with 3 studies included provided information for 6 RoB domains for 2 RCTs, while for the 3^rd^ RCT [37] only one RoB domain was judged in the table – the ‘attrition bias’ domain. In the text the authors wrote that this trial overall had unclear RoB as information were not available.

In the **third group** there was one Cochrane review with one included RCT without 'other bias' domain, and one included RCT with only judgment, without comment [38].

In the **fourth group** there was one Cochrane review where RoB table was completely missing for 6 included RCTs [39]. The authors wrote in the body of the manuscript that these 6 RCTs were reported only as conference abstract and therefore they did not assess RoB for those studies, which resulted in blank rows in Figure 2 that presents RoB summary with review authors' judgements about each risk of bias item for each included study.

*Reviews with both ‘other bias’ domain and additional non-standard domain(s) for other bias in RoB tables*

Nine Cochrane reviews [40-48] had both ‘other bias’ domain and additional non-standard domain(s) for other bias in RoB tables. Those Cochrane reviews used from 1 to 4 additional non-standard domains; 18 total.

*Reviews that consistently did not use support for judgment or they used non-standard judgments*

We found 9 Cochrane reviews that consistently did not use supporting explanations for judgment or they used non-standard judgments. In 5 Cochrane reviews authors used judgments low, high or unclear RoB, but without comments as support for judgment. In one Cochrane review [49] all trials were marked with unclear risk of other bias without any comment as support for judgment. In four Cochrane reviews [50-53] all trials were marked with low risk of other bias without any comment as support for judgment. We also found 4 Cochrane reviews that did not have judgments low-high-unclear, but different kinds of judgments. One Cochrane review had judgments yes/no without supporting comments [54]; two Cochrane reviews had judgments yes, no or unclear, with supporting comments [55, 56] and there was one Cochrane review with judgments A-adequate and B-unclear [57].

**References**

[1] Bennett MH, Lehm JP, Jepson N. Hyperbaric oxygen therapy for acute coronary syndrome. The Cochrane database of systematic reviews. 2015:CD004818.

[2] Taylor-Robinson DC, Maayan N, Soares-Weiser K, Donegan S, Garner P. Deworming drugs for soil-transmitted intestinal worms in children: effects on nutritional indicators, haemoglobin, and school performance. The Cochrane database of systematic reviews. 2015:CD000371.

[3] Pena-Rosas JP, De-Regil LM, Garcia-Casal MN, Dowswell T. Daily oral iron supplementation during pregnancy. The Cochrane database of systematic reviews. 2015:CD004736.

[4] Murdin L, Hussain K, Schilder AG. Betahistine for symptoms of vertigo. The Cochrane database of systematic reviews. 2016:CD010696.

[5] Thomas RE, Jefferson T, Lasserson TJ. Influenza vaccination for healthcare workers who care for people aged 60 or older living in long-term care institutions. The Cochrane database of systematic reviews. 2016:CD005187.

[6] Cahill K, Lindson-Hawley N, Thomas KH, Fanshawe TR, Lancaster T. Nicotine receptor partial agonists for smoking cessation. The Cochrane database of systematic reviews. 2016:CD006103.

[7] Tzortziou Brown V, Underwood M, Mohamed N, Westwood O, Morrissey D. Professional interventions for general practitioners on the management of musculoskeletal conditions. The Cochrane database of systematic reviews. 2016:CD007495.

[8] Lai NM, Taylor JE, Tan K, Choo YM, Ahmad Kamar A, Muhamad NA. Antimicrobial dressings for the prevention of catheter-related infections in newborn infants with central venous catheters. The Cochrane database of systematic reviews. 2016;3:CD011082.

[9] Bruschettini M, Romantsik O, Zappettini S, Banzi R, Ramenghi LA, Calevo MG. Antithrombin for the prevention of intraventricular hemorrhage in very preterm infants. The Cochrane database of systematic reviews. 2016;3:CD011636.

[10] Semlitsch T, Jeitler K, Berghold A, Horvath K, Posch N, Poggenburg S, et al. Long-term effects of weight-reducing diets in people with hypertension. The Cochrane database of systematic reviews. 2016;3:CD008274.

[11] Sankar MJ, Sankar J, Mehta M, Bhat V, Srinivasan R. Anti-vascular endothelial growth factor (VEGF) drugs for treatment of retinopathy of prematurity. The Cochrane database of systematic reviews. 2016;2:CD009734.

[12] Wilkinson D, Andersen C, O'Donnell CP, De Paoli AG, Manley BJ. High flow nasal cannula for respiratory support in preterm infants. The Cochrane database of systematic reviews. 2016;2:CD006405.

[13] Huttner FJ, Fitzmaurice C, Schwarzer G, Seiler CM, Antes G, Buchler MW, et al. Pylorus-preserving pancreaticoduodenectomy (pp Whipple) versus pancreaticoduodenectomy (classic Whipple) for surgical treatment of periampullary and pancreatic carcinoma. The Cochrane database of systematic reviews. 2016;2:CD006053.

[14] Ren Q, Yan X, Zhou Y, Li WX. Periodontal therapy as adjunctive treatment for gastric Helicobacter pylori infection. The Cochrane database of systematic reviews. 2016;2:CD009477.

[15] Coren E, Hossain R, Pardo Pardo J, Bakker B. Interventions for promoting reintegration and reducing harmful behaviour and lifestyles in street-connected children and young people. The Cochrane database of systematic reviews. 2016:CD009823.

[16] Singh N, Halliday HL, Stevens TP, Suresh G, Soll R, Rojas-Reyes MX. Comparison of animal-derived surfactants for the prevention and treatment of respiratory distress syndrome in preterm infants. The Cochrane database of systematic reviews. 2015:CD010249.

[17] Gertler P, Tate RL, Cameron ID. Non-pharmacological interventions for depression in adults and children with traumatic brain injury. The Cochrane database of systematic reviews. 2015:CD009871.

[18] Simancas-Racines D, Osorio D, Marti-Carvajal AJ, Arevalo-Rodriguez I. Leukoreduction for the prevention of adverse reactions from allogeneic blood transfusion. The Cochrane database of systematic reviews. 2015:CD009745.

[19] De Sutter AI, Saraswat A, van Driel ML. Antihistamines for the common cold. The Cochrane database of systematic reviews. 2015:CD009345.

[20] Spittle A, Orton J, Anderson PJ, Boyd R, Doyle LW. Early developmental intervention programmes provided post hospital discharge to prevent motor and cognitive impairment in preterm infants. The Cochrane database of systematic reviews. 2015:CD005495.

[21] Brodlie M, Gupta A, Rodriguez-Martinez CE, Castro-Rodriguez JA, Ducharme FM, McKean MC. Leukotriene receptor antagonists as maintenance and intermittent therapy for episodic viral wheeze in children. The Cochrane database of systematic reviews. 2015:CD008202.

[22] Birks JS, Grimley Evans J. Rivastigmine for Alzheimer's disease. The Cochrane database of systematic reviews. 2015:CD001191.

[23] Matthews A, Haas DM, O'Mathuna DP, Dowswell T. Interventions for nausea and vomiting in early pregnancy. The Cochrane database of systematic reviews. 2015:CD007575.

[24] Lawrie TA, Kulier R, Nardin JM. Techniques for the interruption of tubal patency for female sterilisation. The Cochrane database of systematic reviews. 2015:CD003034.

[25] Visvanathan A, Dennis M, Whiteley W. Parenteral fluid regimens for improving functional outcome in people with acute stroke. The Cochrane database of systematic reviews. 2015:CD011138.

[26] Mocellin S, McCulloch P, Kazi H, Gama-Rodrigues JJ, Yuan Y, Nitti D. Extent of lymph node dissection for adenocarcinoma of the stomach. The Cochrane database of systematic reviews. 2015:CD001964.

[27] Lewis SR, Nicholson A, Smith AF, Alderson P. Alpha-2 adrenergic agonists for the prevention of shivering following general anaesthesia. The Cochrane database of systematic reviews. 2015:CD011107.

[28] Amarasena IU, Chatterjee S, Walters JA, Wood-Baker R, Fong KM. Platinum versus non-platinum chemotherapy regimens for small cell lung cancer. The Cochrane database of systematic reviews. 2015:CD006849.

[29] Pouwer AW, Farquhar C, Kremer JA. Long-acting FSH versus daily FSH for women undergoing assisted reproduction. The Cochrane database of systematic reviews. 2015:CD009577.

[30] Whitworth M, Bricker L, Mullan C. Ultrasound for fetal assessment in early pregnancy. The Cochrane database of systematic reviews. 2015:CD007058.

[31] Marti-Carvajal AJ, Simancas-Racines D, Pena-Gonzalez BS. Prolonged storage of packed red blood cells for blood transfusion. The Cochrane database of systematic reviews. 2015:CD009330.

[32] Garner B, Phillips LJ, Bendall S, Hetrick SE. Antiglucocorticoid and related treatments for psychosis. The Cochrane database of systematic reviews. 2016:CD006995.

[33] Frost JA, Webster KE, Bryant A, Morrison J. Lymphadenectomy for the management of endometrial cancer. The Cochrane database of systematic reviews. 2015:CD007585.

[34] van der Linden M, Buckingham K, Farquhar C, Kremer JA, Metwally M. Luteal phase support for assisted reproduction cycles. The Cochrane database of systematic reviews. 2015:CD009154.

[35] Aziz Z, Cullum N. Electromagnetic therapy for treating venous leg ulcers. The Cochrane database of systematic reviews. 2015:CD002933.

[36] Ieran M, Zaffuto S, Bagnacani M, Annovi M, Moratti A, Cadossi R. Effect of low frequency pulsing electromagnetic fields on skin ulcers of venous origin in humans: a double-blind study. Journal of orthopaedic research : official publication of the Orthopaedic Research Society. 1990;8:276-82.

[37] Fayallah EA, Hemida RA, Gamal AM, Abd Elhady E, Anwar KI, Nada NA, et al. Pretreatment study of P53 overexpression for selection of candidates for pelvic lymphadenectomy in clinical stage I endometrial carcinoma: a randomized-controlled study. Archives of gynecology and obstetrics. 2011;283:617-22.

[38] Taverny G, Mimouni Y, LeDigarcher A, Chevalier P, Thijs L, Wright JM, et al. Antihypertensive pharmacotherapy for prevention of sudden cardiac death in hypertensive individuals. The Cochrane database of systematic reviews. 2016;3:CD011745.

[39] Cody JD, Hodson EM. Recombinant human erythropoietin versus placebo or no treatment for the anaemia of chronic kidney disease in people not requiring dialysis. The Cochrane database of systematic reviews. 2016:CD003266.

[40] Desborough M, Sandu R, Brunskill SJ, Doree C, Trivella M, Montedori A, et al. Fresh frozen plasma for cardiovascular surgery. The Cochrane database of systematic reviews. 2015:CD007614.

[41] Pillai Riddell RR, Racine NM, Gennis HG, Turcotte K, Uman LS, Horton RE, et al. Non-pharmacological management of infant and young child procedural pain. The Cochrane database of systematic reviews. 2015:CD006275.

[42] Paley CA, Johnson MI, Tashani OA, Bagnall AM. Acupuncture for cancer pain in adults. The Cochrane database of systematic reviews. 2015:CD007753.

[43] Hayward G, Thompson MJ, Perera R, Del Mar CB, Glasziou PP, Heneghan CJ. Corticosteroids for the common cold. The Cochrane database of systematic reviews. 2015:CD008116.

[44] Crighton GL, Estcourt LJ, Wood EM, Trivella M, Doree C, Stanworth S. A therapeutic-only versus prophylactic platelet transfusion strategy for preventing bleeding in patients with haematological disorders after myelosuppressive chemotherapy or stem cell transplantation. The Cochrane database of systematic reviews. 2015:CD010981.

[45] Gluud LL, Dam G, Les I, Cordoba J, Marchesini G, Borre M, et al. Branched-chain amino acids for people with hepatic encephalopathy. The Cochrane database of systematic reviews. 2015:CD001939.

[46] Weiss NS, Nahuis M, Bayram N, Mol BW, Van der Veen F, van Wely M. Gonadotrophins for ovulation induction in women with polycystic ovarian syndrome. The Cochrane database of systematic reviews. 2015:CD010290.

[47] Furlan AD, Giraldo M, Baskwill A, Irvin E, Imamura M. Massage for low-back pain. The Cochrane database of systematic reviews. 2015:CD001929.

[48] Hooper L, Abdelhamid A, Bunn D, Brown T, Summerbell CD, Skeaff CM. Effects of total fat intake on body weight. The Cochrane database of systematic reviews. 2015:CD011834.

[49] Rezk E, Nofal YH, Hamzeh A, Aboujaib MF, AlKheder MA, Al Hammad MF. Steroids for symptom control in infectious mononucleosis. The Cochrane database of systematic reviews. 2015:CD004402.

[50] Elbers RG, Verhoef J, van Wegen EE, Berendse HW, Kwakkel G. Interventions for fatigue in Parkinson's disease. The Cochrane database of systematic reviews. 2015:CD010925.

[51] Anabrees J, Shah VS, AlOsaimi A, AlFaleh K. Glycerin laxatives for prevention or treatment of feeding intolerance in very low birth weight infants. The Cochrane database of systematic reviews. 2015:CD010464.

[52] Liet JM, Ducruet T, Gupta V, Cambonie G. Heliox inhalation therapy for bronchiolitis in infants. The Cochrane database of systematic reviews. 2015:CD006915.

[53] Tudor KI, Tudor M, McCleery J, Car J. Endoscopic third ventriculostomy (ETV) for idiopathic normal pressure hydrocephalus (iNPH). The Cochrane database of systematic reviews. 2015:CD010033.

[54] Riera R, Porfirio GJ, Torloni MR. Alemtuzumab for multiple sclerosis. The Cochrane database of systematic reviews. 2016;4:CD011203.

[55] Marcano Belisario JS, Jamsek J, Huckvale K, O'Donoghue J, Morrison CP, Car J. Comparison of self-administered survey questionnaire responses collected using mobile apps versus other methods. The Cochrane database of systematic reviews. 2015:MR000042.

[56] Marusic A, Wager E, Utrobicic A, Rothstein HR, Sambunjak D. Interventions to prevent misconduct and promote integrity in research and publication. The Cochrane database of systematic reviews. 2016;4:MR000038.

[57] Bartels EM, Juhl CB, Christensen R, Hagen KB, Danneskiold-Samsoe B, Dagfinrud H, et al. Aquatic exercise for the treatment of knee and hip osteoarthritis. The Cochrane database of systematic reviews. 2016;3:CD005523.
